# Tick defensin γ-core reduces *Fusarium graminearum* growth and abrogates mycotoxins production with high efficiency

**DOI:** 10.1038/s41598-021-86904-w

**Published:** 2021-04-12

**Authors:** Valentin Leannec-Rialland, Alejandro Cabezas-Cruz, Vessela Atanasova, Sylvain Chereau, Nadia Ponts, Miray Tonk, Andreas Vilcinskas, Nathalie Ferrer, James J. Valdés, Florence Richard-Forget

**Affiliations:** 1 Université de Bordeaux, INRAE, Mycology and Food Safety (MycSA), 33882 Villenave d’Ornon, France; 2 Anses, INRAE, Ecole Nationale Vétérinaire D’Alfort, UMR BIPAR, Laboratoire de Santé Animale, 94700 Maisons-Alfort, France; 3 INRAE, Mycology and Food Safety (MycSA), 33882 Villenave d’Ornon, France; 4 Institute for Insect Biotechnology, Justus Liebig University of Giessen, Heinrich-Buff-Ring 26-32, 35392 Giessen, Germany; 5 LOEWE Centre for Translational Biodiversity Genomics (LOEWE-TBG), Senckenberganlage 25, 60325 Frankfurt, Germany; 6 Department of Bioresources, Fraunhofer Institute for Molecular Biology and Applied Ecology, Ohlebergsweg 12, 35392 Giessen, Germany; 7 Faculty of Science, University of South Bohemia, Branišovská 1160/31, 37005 České Budějovice, Czech Republic; 8 Institute of Parasitology, Biology Centre, Czech Academy of Sciences, Branišovská 1160/31, 37005 České Budějovice, Czech Republic; 9 Department of Virology, Veterinary Research Institute, Hudcova 70, 62100 Brno, Czech Republic

**Keywords:** Peptides, Plant sciences, Antimicrobials, Fungi, Molecular modelling

## Abstract

*Fusarium graminearum* is a major fungal pathogen affecting crops of worldwide importance. *F. graminearum* produces type B trichothecene mycotoxins (TCTB), which are not fully eliminated during food and feed processing. Therefore, the best way to minimize TCTB contamination is to develop prevention strategies. Herein we show that treatment with the reduced form of the γ-core of the tick defensin DefMT3, referred to as TickCore3 (TC3), decreases *F. graminearum* growth and abrogates TCTB production. The oxidized form of TC3 loses antifungal activity, but retains anti-mycotoxin activity. Molecular dynamics show that TC3 is recruited by specific membrane phospholipids in *F. graminearum* and that membrane binding of the oxidized form of TC3 is unstable. Capping each of the three cysteine residues of TC3 with methyl groups reduces its inhibitory efficacy. Substitutions of the positively-charged residues lysine (Lys) 6 or arginine 7 by threonine had the highest and the lesser impact, respectively, on the anti-mycotoxin activity of TC3. We conclude that the binding of linear TC3 to *F. graminearum* membrane phospholipids is required for the antifungal activity of the reduced peptide. Besides, Lys6 appears essential for the anti-mycotoxin activity of the reduced peptide. Our results provide foundation for developing novel and environment-friendly strategies for controlling *F. graminearum*.

## Introduction

*Fusarium graminearum* is the major causal agent of two devastating diseases affecting cereal-growing areas around the world. Fusarium head blight (FHB) affects small grain cereals including wheat, oat, barley, rice and rye, a Gibberella ear rot (GER) has a major impact on maize production. In addition to cause grain yield losses^1,2^, *F. graminearum* can produce mycotoxins of the family type B trichothecenes (TCTB), including deoxynivalenol (DON) and its 15- or 3-acetylated forms (15- and 3-ADON), that constitute important food contaminants. DON toxicity has been well-documented and represents a public health concern^3^. To protect consumers, maximum admissible DON content for cereals and maize-based food were set by the European Commission in June 2005 (EC No856/2005) and revised in July 2007 (EC No1126/2007). DON is resistant to biodegradation and food processing and currently, there is no suitable detoxification procedure available. As a consequence, the occurrence of TCTB in cereal-derived products cannot be reduced by post-harvest treatments. Although good agricultural practices have been proposed to reduce the impact of *Fusarium* and DON contamination on cereal grains, they are not always efficient to guarantee the compliance with the safety regulations. Thus, the development of innovative sustainable and environment-friendly solutions to reduce contaminations of cereals with mycotoxins produced by *F. graminearum* is particularly encouraged.

Ticks are important vectors of pathogens affecting human and animal health worldwide^4^. Ticks carry a high diversity of pathogenic and non-pathogenic microorganisms including bacteria, viruses, protozoa, helminths and fungi^4–6^. Ticks develop a vast array of humoral and cellular immune reactions in response to microbial challenge^7^. Defensins, a family of antimicrobial peptides (AMP), are major effector molecules of tick humoral immunity^7^. Tick defensins have a wide spectrum of antimicrobial activity against gram-negative and gram-positive bacteria, but also against eukaryotes such as fungi and apicomplexan parasites^8–11^. Similar to plant defensins, tick defensins inhibit the growth of *F. graminearum*^8,12,13^. Previous studies showed that the **γ**-cores of *Ixodes ricinus* defensins, DefMT3 and DefMT6, are potent antifungal agents that inhibit *F. graminearum* germination in a dose-dependent manner^9^. In this study, we asked whether tick defensin **γ**-cores would inhibit TCTB production by *F. graminearum* and aimed to identify the structural determinants of such activity. Our study, focusing on the **γ**-core of DefMT3, showed that Cys cyclization is not necessary for the antifungal and anti-mycotoxin activity of tick **γ**-cores. Furthermore, we demonstrated the key role of positively charged amino acids for both types of activity.

## Material and methods

### Synthesis of tick γ-core TickCore3

TickCore3 (TC3) is the **γ**-core of the *Ixodes ricinus* defensin DefMT3 (GenBank accession number: JAA71488)^9^. Peptide synthesis was commissioned to Pepmic (Suzhou, China) that uses solid-phase peptide synthesis (SPPS) to obtain highly-pure peptides as previously described^10^. Briefly, peptide synthesis was performed using 2-chlorotrityl chloride resin as the solid support, using the base labile 9-fluorenyl-methyloxy-carbonyl (Fmoc) as protecting group. Amino acids were protected as follows: Fmoc-Cys(Trt)-OH, Fmoc-Gly-OH, Fmoc-Asn(Trt)-OH, Fmoc-Phe-OH, Fmoc-Leu-OH, Fmoc-Lys(Boc)-OH, Fmoc-Arg(Pbf)-OH, Fmoc-Thr(tBu)-OH, Fmoc-Ile-OH and Fmoc-Val-OH. All the peptide sequences were synthesized according to the principles of SPPS. High Performance Liquid Chromatography (HPLC)-grade Fmoc chloride (Fmoc-Cl)-protected amino acid-based peptide chemistry was used with standard peptide chemistry coupling protocols. Peptides were purified by reverse phase HPLC and peptide sequence was confirmed by electrospray mass spectroscopy (ESI–MS) using a mass spectrometer LCMS-2020 (Shimadzu, Kyoto, Japan). Oxidization was achieved using a folding buffer containing 1 M urea, 100 mM Tris (pH 8.0), 1.5 mM oxidized glutathione, 0.75 mM reduced glutathione and 10 mM methionine. Oxidation of Cys residues was confirmed by Ellman reagent reaction and sulfide bond formation was characterized by ESI–MS on a mass spectrometer LCMS-2020 (Shimadzu, Kyoto, Japan). The peptide TC3Ox was synthetized as TC3, but synthesis was followed by oxidation using folding buffer. The peptides TC3-CH3-123, TC3-CH3-1, TC3-CH3-2 and TC3-CH3-3, TC3-CH3-1Ox, TC3-CH3-2Ox and TC3-CH3-3Ox were synthesized as above but Fmoc-Cys(Me)-OH was added instead of Fmoc-Cys(Trt)-OH and the synthesis of TC3-CH3-1Ox, TC3-CH3-2Ox and TC3-CH3-3Ox was followed by oxidation using folding buffer. Capping the sulfur (S) of the thiol group of all Cys prevents Cys cyclization (*i.e.,* no disulfide bridge can be formed). Conversely, capping the S of the thiol group of individual Cys followed by oxidation directs the cyclization of the reduced Cys (*i.e.,* those that were not capped). Four peptides were synthetized as TC3 with amino acid substitutions, referred as TC3-K6T (Lys6 substituted by Thr), TC3-R7T (Arg7 substituted by Thr), TC3-K13T (Lys13 substituted by Thr) and TC3-K14T (Lys 14 substituted by Thr). The sequences of TC3 and all TC3 variants are available in Table 1.

**Table 1:** List of peptides synthetized and used in this study.

Peptide names	Sequence^a^	Synthesis modification
TC3	CGNFLKRTCICVKK	Reduced
TC3Ox	CGNFLKRTCICVKK	Oxidation
TC3-CH3-123	C(CH_3_)GNFLKRTC(CH_3_)IC(CH_3_)VKK	Methylation
TC3-CH3-1	C(CH_3_)GNFLKRTCICVKK	Methylation
TC3-CH3-2	CGNFLKRTC(CH_3_)ICVKK	Methylation
TC3-CH3-3	CGNFLKRTCIC(CH_3_)VKK	Methylation
TC3-CH3-1Ox	C(CH_3_)GNFLKRTCICVKK	Methylation followed by oxidation
TC3-CH3-2Ox	CGNFLKRTC(CH_3_)ICVKK	Methylation followed by oxidation
TC3-CH3-3Ox	CGNFLKRTCIC(CH_3_)VKK	Methylation followed by oxidation
TC3-K6T	CGNFL**T**RTCICVKK	Amino acid substitution
TC3-R7T	CGNFLK**T**TCICVKK	Amino acid substitution
TC3-K13T	CGNFLKRTCICV**T**K	Amino acid substitution
TC3-K14T	CGNFLKRTCICVK**T**	Amino acid substitution

^a^Amino acids substitutions are underlined and in bold letters.

### Classical molecular dynamics

#### TC3 and membrane structures

The tertiary structure of the linearized TC3 peptide was constructed in silico by truncating the predicted DefMT3 *Ixodes ricinus* salivary defensin from our previous study^9^. Both termini of the TC3 were capped with an acetyl group (amine terminus) and a N-methyl amide group (carboxyl terminus). The electrostatic potential of TC3 was generated using tools from the Schrodinger’s Maestro molecular software package, release 2020–4^14^. The CHARMM-GUI automated server^15^ was used to construct the heterogenous bilayer membrane. The upper leaflet of the membrane bilayer consisted of 62 phospholipids [32 phosphatidylcholine (POPC), 14 phosphatidylethanolamine (POPE), 6 phosphatidylinositol (POPI), 4 phosphatidylserine (POPS), 2 phosphatidic acid (POPA), 2 phosphatidylglycerol (POPG), and tetraoleoyl cardiolipin 2 (TOCL)]. The lower leaflet consisted of 64 phospholipids (36 POPC and 28 POPE). The heterogenous bilayer membrane therefore consisted of 126 phospholipids. The TC3 and membrane systems were then prepared separately and the hydrogen-bond network was optimized using the Schrodinger’s Maestro Protein Preparation Wizard^16^. After preparation and optimization, a global minimization was performed using Maestro default settings to remove any steric clashes.

#### All-atom molecular simulation protocol

The optimized TC3 peptide and bilayer membrane were oriented separately in an orthorhombic box (40 Å × 40 Å × 40 Å and 64 Å × 64 Å × 130 Å, respectively) with a TIP3P explicit model, neutralized and salted with 0.15 M NaCl. The force fields used to parameterize the two systems were, TIP3P CHARMM^17^ for the solvent, AMBER99SB-ILDN^18–20^ for the TC3 peptide, and CHARMM36^21^ for the membrane and ions. The program CGenFF^22,23^, based on CHARMM36^21^, was used to retrieve force field parameters for phospholipids POPI and TOCL. Classical molecular dynamics (MD) calculations were performed using Desmond^24^. Semi-isotropic conditions for MD were used under an NPT ensemble coupled with a Nose–Hoover thermostat^25^ and Martyna–Tobias–Klein barostat^26^. The temperature was set at 37 °C with a RESPA^27^ integrator at an inner time step of 2.5 fs. All MD calculations were conducted using a GPU-accelerated (*i.e.,* graphics card) workstation.

The phospholipid bilayer membrane was first equilibrated using the Desmond^24^ protocol provided by the CHARMM-GUI automated server^15^. The final CHARMM-GUI membrane production frame generated and the TC3 were separately equilibrated for 4 μs and 1 μs, respectively, using the MD protocol above. The final frame from the TC3 and membrane equilibration was used as the starting conformations for the combined peptide-membrane system. The TC3 was positioned 30 Å from its residue α-carbons to the phospholipid phosphorus atoms of the upper membrane surface and was equilibrated, as before, for 100 ns in an orthorhombic box 64 Å × 64 Å × 130 Å. Production MD calculations for analyses were then conducted for 1 μs using the last equilibrated frame under the same above protocol except using an NVT ensemble (where the volume is kept constant, not the pressure). The MD calculations for the cysteine oxidized form of TC3 (TC3Ox) was conducted using the same aforementioned protocol. The MD calculations were analysed using the Schrodinger’s Maestro software package^14^.

### Mycotoxin production inhibition assay

#### Fusarium strain and culture conditions

The *F. graminearum* CBS185.32 strain (Westerdijk Institute, The Netherlands) was selected for its capacity to produce high concentrations of DON and 15-ADON and was used throughout the study. Fungal culture was maintained at 4 °C on Potato Dextrose Agar (PDA) (Difco, Le Ponts de Claix, France) slants under mineral oil. Conidia were prepared by inoculating agar plugs in CMC medium (15 g/L carboxylmethyl cellulose, 1 g/L yeast extract, 0.5 g/L MgSO_4_.7H_2_O, 1 g/L NH_4_NO_3_, 1 g/L KH_2_PO_4_), incubating at 150 rpm and 25 °C for three to five days, and harvesting by filtration through Sefar Nitex 03–100 (SEFAR AG, Heiden, Switzerland). Liquid-culture experiments were performed in 24-well static plates. Each well containing 2 mL of a mycotoxin synthetic (MS) medium (20 g/L glucose, 0.5 g/L KH2PO4, 0.6 g/L K_2_HPO_4_, 17 mg/L MgSO4, 1 g/L (NH4)2SO4, 0.1 mL/L Vogel mineral salts solution^28^) prepared as previously described^29^, supplemented or not with TC3 or its variants at three concentrations (12.5, 25 and 50 µM), was inoculated with 2 × 10^4^ spores/mL. Fungal liquid cultures were incubated at 25 °C in the dark for 10 days. Following incubation, mycelia were recovered by centrifugation and fungal biomass was measured by weighing the mycelia after 48 h of freeze-drying (Flexi-Dry, Oerlikon Leybold, Germany). Culture media were stored at -20 °C until TCTB analysis. All peptides were tested at three concentrations 12.5, 25 and 50 µM. Five repetitions were made for each condition. Controls using TC3-free control media and non-inoculated control media were included.

#### Extraction and analysis of TCTB

The procedure was adapted from a previously published procedure^29^. Briefly, 1.5 mL sample of culture medium was extracted with 3 mL of ethyl acetate. A volume of 2.5 mL of the organic phase was evaporated to dryness at 45 °C under nitrogen flux. Dried samples were dissolved in 200 µL of methanol/water (1/1, v/v) and filtered through a 0.2 µm filter before analysis. TCTB were quantified by HPLC–DAD using an Agilent Technologies 1100 series liquid chromatograph equipped with an auto sampler system, an Agilent photodiode array detector (DAD) and the ChemStation chromatography manager software (Agilent, France). Separation was achieved on a Kinetex XB-C18 100 Å column (4.6 × 150 mm, 2.6 μm) (Phenomenex, France) maintained at 45 °C. The mobile phase consisted of water acidified with ortho-phosphoric acid to pH 2.6 (solvent A) and acetonitrile (solvent B). The flow was kept at 1 mL min^−1^. The injection volume was set to 5 μL. TCTB were separated using a gradient elution as follows: 7–30% B in 10 min, 30–90% B in 5 min, 90% B for 5 min, 90–7% B for 2 min, 7% B for 5 min. The UV–Vis spectra were recorded from 190 to 400 nm and peak areas were measured at 230 nm. Quantification was performed by external calibration with standard solutions (Romer Labs, Austria). Toxin yields were expressed in μg g^−1^ of fungal dry biomass.

### Statistical analyses

All presented values are mean ± standard deviation including five biological replications. Since the data were non-normally distributed (Shapiro–Wilk normality test), the Kruskal–Wallis one-way analysis was used, with mean comparisons performed using the Mann–Whitney U test. Statistical analysis was conducted with R^30^ and figures were produced using the package ggplot2^31^. The statistical thresholds *p* = 0.05 (*), *p* = 0.01 (**) and *p* = 0.005 (***) were used throughout the study.

## Results

### Linear TC3 is a strong inhibitor of TCTB production by *F. graminearum*

**Figure 1:**
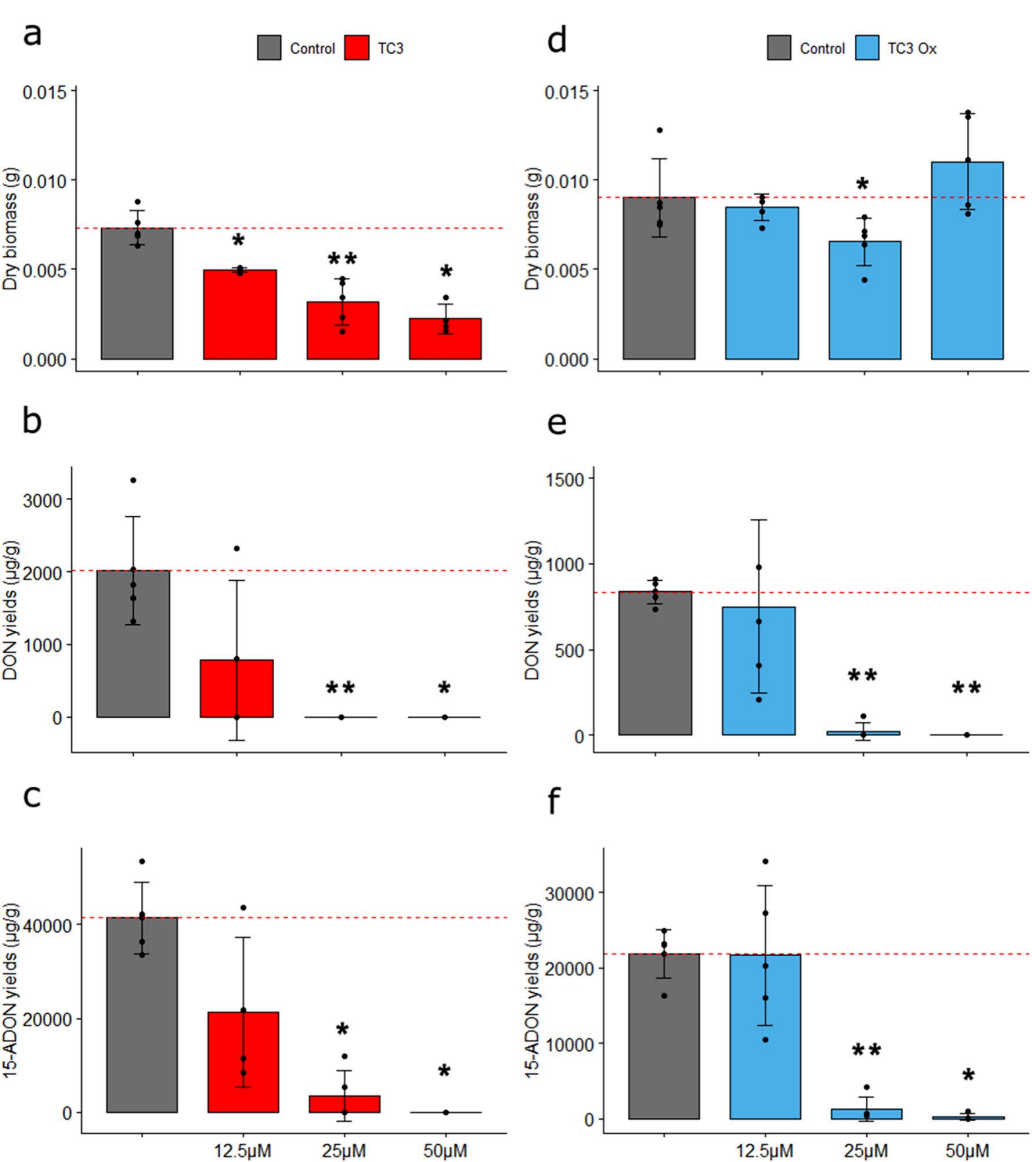
Antifungal and anti-mycotoxin activity of natural and oxidized TC3. Effect of reduced TC3 (**a**–**c**) and oxidized TC3 (**d**–**f**) at 12.5, 25 and 50 µM on the fungal biomass weight of *F. graminearum* (**a**, **d**) and on the production of DON (**b**, **e**) and 15-ADON (**c**, **f**) in 10-day-old broths. Significant differences are labelled (*p < 0.05, **p < 0.01 and ***p < 0.005).

*F. graminearum* cultures were supplemented or not with the peptide TC3. A significant dose-dependent inhibition of fungal growth was registered under all tested TC3 concentrations compared with control samples (Fig. 1a). Concerning mycotoxin production, 15-ADON was the more abundant TCTB detected in the liquid media (Fig. 1b,c). In control samples, production of 15-ADON (41,347 ± 7622 µg/g) was 20 times higher than that of DON (2014 ± 742 µg/g). Supplementation with 25 and 50 µM TC3 reduced both DON (Fig. 1b) and 15-ADON (Fig. 1c) synthesis to trace amounts. However, only a non-significant reduction of DON (Fig. 1b) and 15-ADON (Fig. 1c) was observed with 12.5 µM TC3. Compared to TC3, the TC3Ox had a reduced effect on the dry fungal biomass (Fig. 1d). However, similar to TC3, the detected amounts of DON (Fig. 1e) and 15-ADON (Fig. 1f) were significantly decreased in the 25 µM and 50 µM TC3Ox-treated samples.

The purity, amino acid sequence and reduction status of the TC3 and TC3Ox cysteine (Cys) residues were evaluated by HPLC and ESI–MS (Supplementary Figure S1). A major peak, representing 95% of the composition of the purified peptide, was observed in the HPLC chromatogram of TC3. The observed molecular weight (MW) of the purified TC3 (*i.e.,* 1613 Da) matched the theoretical MW of TC3 (Supplementary Figure S1) and included the three hydrogen (H^+^, considering that the MW of H^+^ is approximately 1) of the thiol groups of the three Cys. TC3Ox was purified and refolded in folding buffer to obtain the oxidized form of TC3. HPLC analysis of the final purified and oxidized TC3Ox revealed a complex mix of peptides eluted with different retention times (Supplementary Figure S1). The identity of the most abundant peptide, representing 44% of the area, was characterized by ESI–MS and revealed the presence of a peptide with a MW that matches the MW of a homodimer TC3, referred here to as TC3-TC3. The MW of TC3-TC3 having three –S–S– bonds should be approximately 6 Da less than the double of the MW of a monomer of TC3 before the oxidation (*i.e.,* (1613 * 2) − 6 = 3220), resulting from the loss of 6 H^+^ from the six reduced sulfhydryl groups (–SH). This was confirmed using ESI–MS; the observed MW of TC3–TC3 was indeed 3220 Da (Supplementary Figure S1). In addition to the dimer TC3–TC3, three possible disulphide bridge combinations (Cys1–Cys9, Cys1–Cys11 and Cys9–Cys11) could result in three different oxidized monomers of TC3. However, no oxidized TC3 monomer was detected by ESI–MS probably due to the lower representation within the oxidized mix and concentrations below detection thresholds (Supplementary Figure S1).

### Linear TC3 is recruited by specific membrane phospholipids

**Figure 2:**
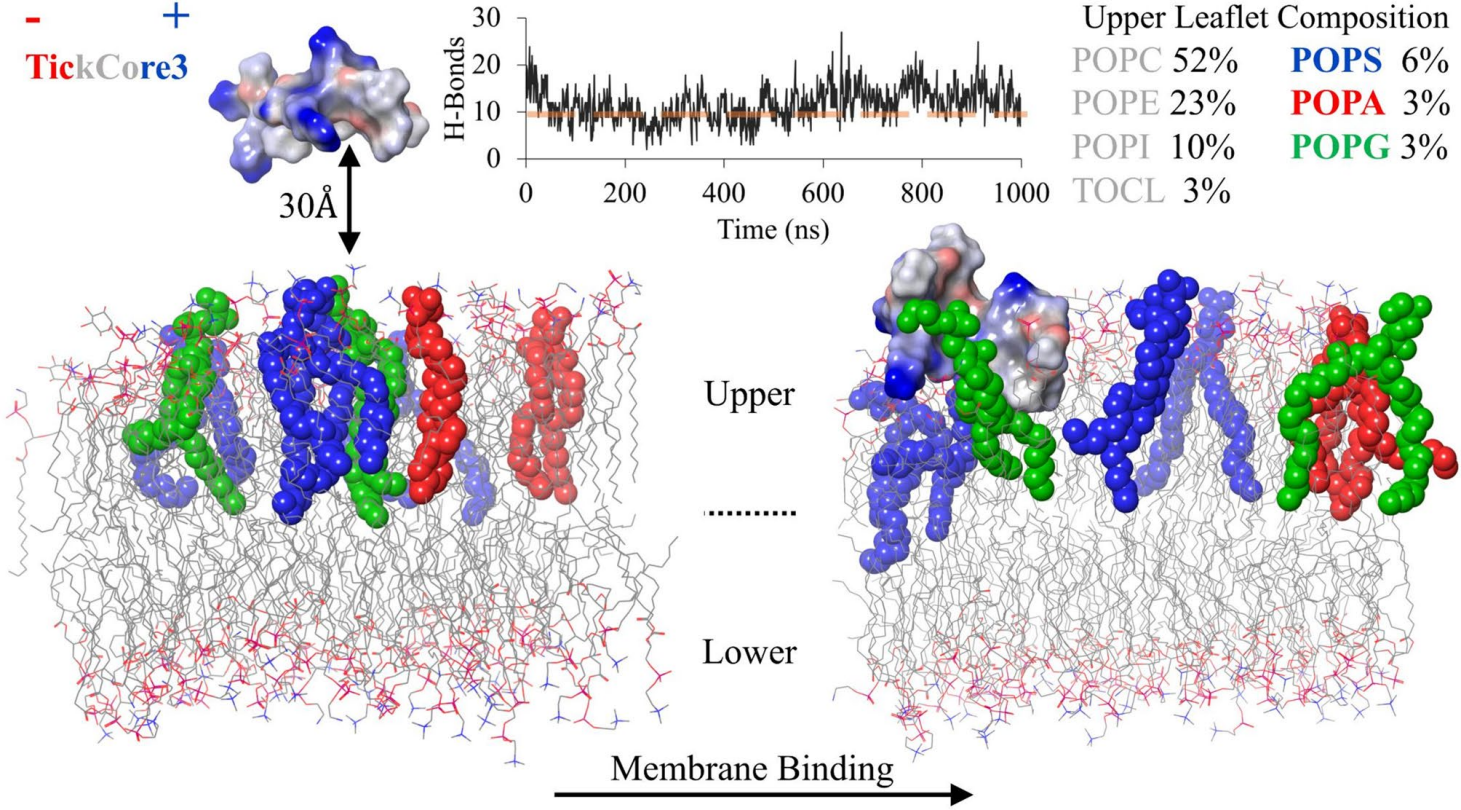
Membrane binding of TickCore3. The TC3 electrostatic potential of its residues are color-labeled as red (negative), grey (neutral) and blue (positive). The TC3 was placed 30 Å—from residue α-carbons to the phospholipid phosphorus atoms of the upper membrane surface. The upper and lower membrane leaflets, before and after 1 μs of MD, are shown with the majority of phospholipids depicted as sticks (grey = carbon, red = oxygen, and blue = nitrogen) (hydrogen atoms not shown). The main phospholipids (POPS, POPA and POPG) forming hydrogen bonds with TC3 are depicted as colored spheres (see legend for color scheme and percent composition). The line graph depicts the number hydrogen bond contacts (H-Bonds; y-axis) throughout the 1 μs of MD (x-axis; in ns). The dashed orange line in the graph is the average H-Bond contacts ($\overline{x} = 9.7$) between the membrane and the TC3Ox.

The in silico TC3 peptide for the MD calculations is a truncated form of the previous predicted tertiary structure of the *I. ricinus* DefMT3^9^. The TC3 electrostatic potential is mainly neutral with four highly positive residues (Lys/Arg) (Fig. 2). To approximate a natural heterogenous membrane, the upper leaflet was based on the actual phospholipid composition of *F. graminearum*^32^ (Fig. 2). The TC3 was positioned with most of its residues facing away from or parallel to the upper membrane leaflet (Fig. 2). After equilibration (100 ns; see Material and Methods), the TC3 was immediately recruited by the upper leaflet. During the 1 μs MD production stage, the hydrogen bond contacts formed between the TC3 and the upper leaflet reduced for the first 500 ns, but later increased after 600 ns (line graph in Fig. 2). The specific upper leaflet phospholipid types that recruit TC3 are POPS, POPA and POPG (color-coded in Fig. 2). Although multiple copies of these phospholipid types were present in the upper leaflet (Fig. 2), TC3 only formed contacts with a single POPS, POPA and/or POPG during the last 400 ns of the 1 μs MD.

**Figure 3:**
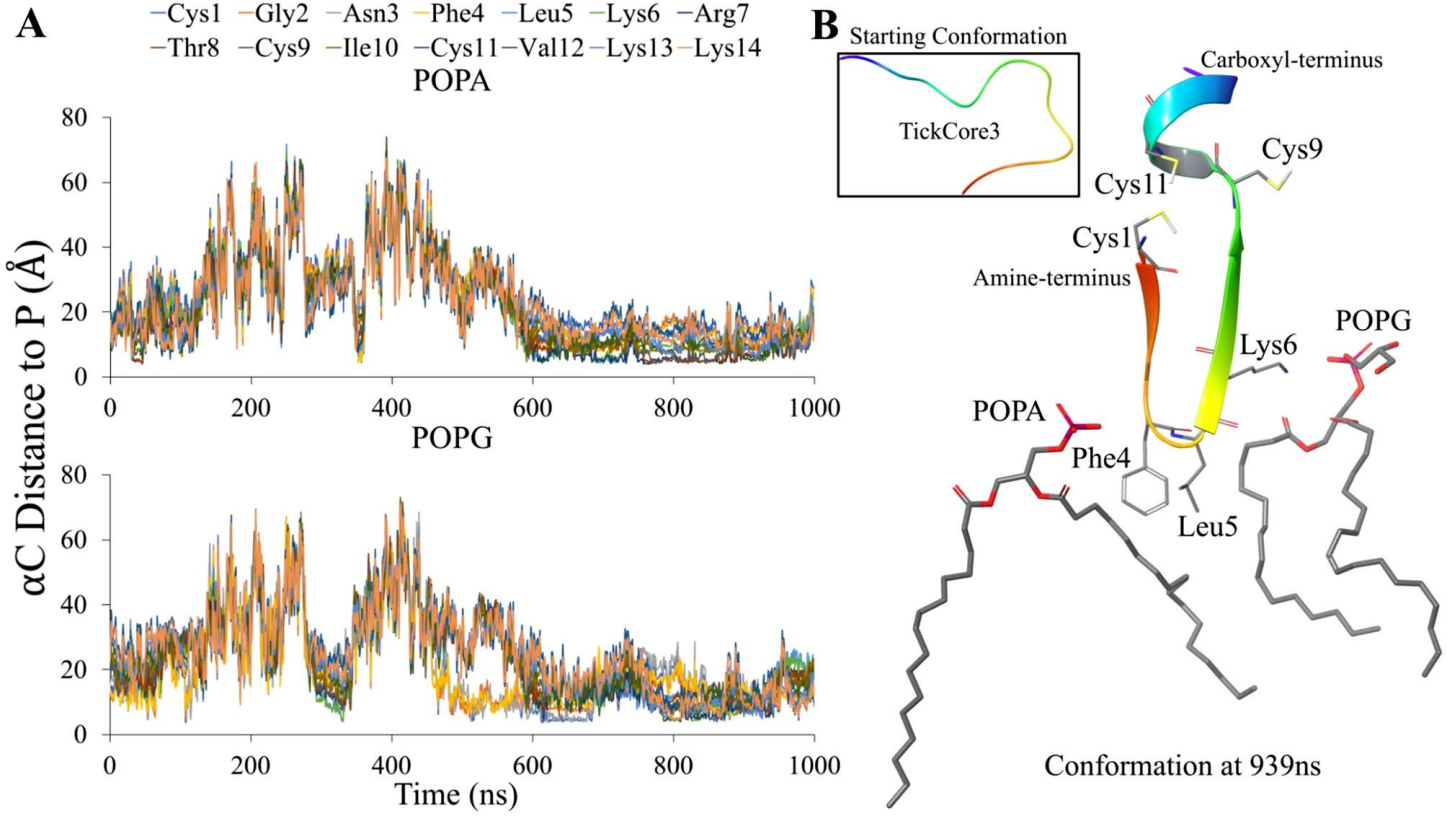
Recruitment of TC3 by POPA and POPG phospholipids. The line graphs (**A**) are the distances measured (y-axis) between the α-carbon (αC) of TC3 residues and the phosphorus (P) atom of POPA (upper) and POPG (lower) throughout the 1 μs of MD (x-axis; in ns). The structural conformation at 0.939 μs of MD (**B**) shows the specific TC3 residues 4–6 that interact with POPA and POPG (hydrogen atoms not shown). The tertiary structure of TC3 also depicts the Cys residues (with hydrogen caps) and is color-coded from the amine-terminus (red) to the carboxyl-terminus (purple).

The TC3 was membrane-bound after equilibration, but, as indicated by the increased distances from POPA and POPG (Fig. 3A), disassociates from the upper leaflet during the first 500 ns. The membrane recruitment of TC3 then stabilizes with POPA and POPG approximately at 600 ns (Fig. 3A). After 600 ns, TC3-POPA average contact distances were between 7–14 Å and 11–15 Å with POPG (Fig. 3A). Association with POPS demonstrated greater distances with TC3 after 600 ns of MD (averaging 18–25 Å; Supplementary Figure S2). Between the two phospholipids, on average, POPA forms closer contacts (< 11 Å) with half of TC3 than POPG, namely with residues Leu5—Ile10 and Val12 (Fig. 3A). The closest average distance, however, for both POPA (6.8 Å) and POPG (11.2 Å), is with TC3 residue Arg7 (Fig. 3A).

The TC3Ox formed an average of 9.7 hydrogen bond contacts with the membrane throughout the 1 μs MD, similarly as the first 500 ns of the TC3 MD calculations (line graph A in Fig. 3). The TC3Ox MD calculations show unstable contacts formed with POPA, POPG and POPS (Supplementary Figure S3A). The TC3Ox secondary structure conformations during the 1 μs MD are dominated by simple turns and coils with helical folding occurring at both peptide termini after ~ 400 ns of MD (Supplementary Figure S3B). The helical folding coincides when TC3Ox momentarily associates with the membrane (Supplementary Figure S3).

Depending on membrane disassociation and association (Fig. 3A and Supplementary Figure S2), TC3 undergoes considerable secondary structure conformations throughout the 1 μs MD (Supplemental Figure S4). The TC3 during the first half of the 1 μs MD is marked by coils and turns with sporadic helices forming throughout residues Cys4—Ile10 (Supplemental Figure S4). These dynamics are quite similar to the folds occurring during the 1 μs MD of TC3Ox frequent disassociation with the membrane (Supplementary Figure S3). Only after 580 ns, while approximating membrane association (Fig. 3A and Supplementary Figure S2), does TC3 fold into more ordered secondary structures (Supplementary Figure S4). The MD timeline demonstrates (Supplementary Figure S4) that TC3 transitions between a simple coil-turn structure (membrane disassociation) to a β-hairpin/α-helix structure (membrane association)—as shown in complex with POPA and POG (Fig. 3B).

### Methylation of Cys reduces the antifungal and anti-mycotoxin activities of TC3

The purity and sequence of all methylated variants of TC3, TC3-CH3-1Ox, TC3-CH3-2Ox and TC3-CH3-3Ox, were confirmed by HPLC and ESI–MS, respectively (Supplementary Figure S5). As shown in the HPLC chromatogram, high purity (*i.e.,* ∼95%) was achieved for the oxidized peptides TC3-CH3-1Ox, TC3-CH3-2Ox and TC3-CH3-3Ox (Supplementary Figure S5). The observed MW (*i.e.,* 1627 Da) of the purified **γ**-cores TC3-CH3-1, TC3-CH3-2 and TC3-CH3-3 before oxidation corresponded with the theoretical MW for each of them (*i.e.,* 1627 Da). The ESI–MS analysis performed after the oxidation protocol confirmed the loss of two H^+^ in each peptide. Quantitative analysis of -SH revealed that no Cys residue was available for reaction with the Ellman’s reagent, indicating, in combination with the ESI–MS results, that all the non-capped Cys residues were involved in intramolecular disulphide linkages.

**Figure 4:**
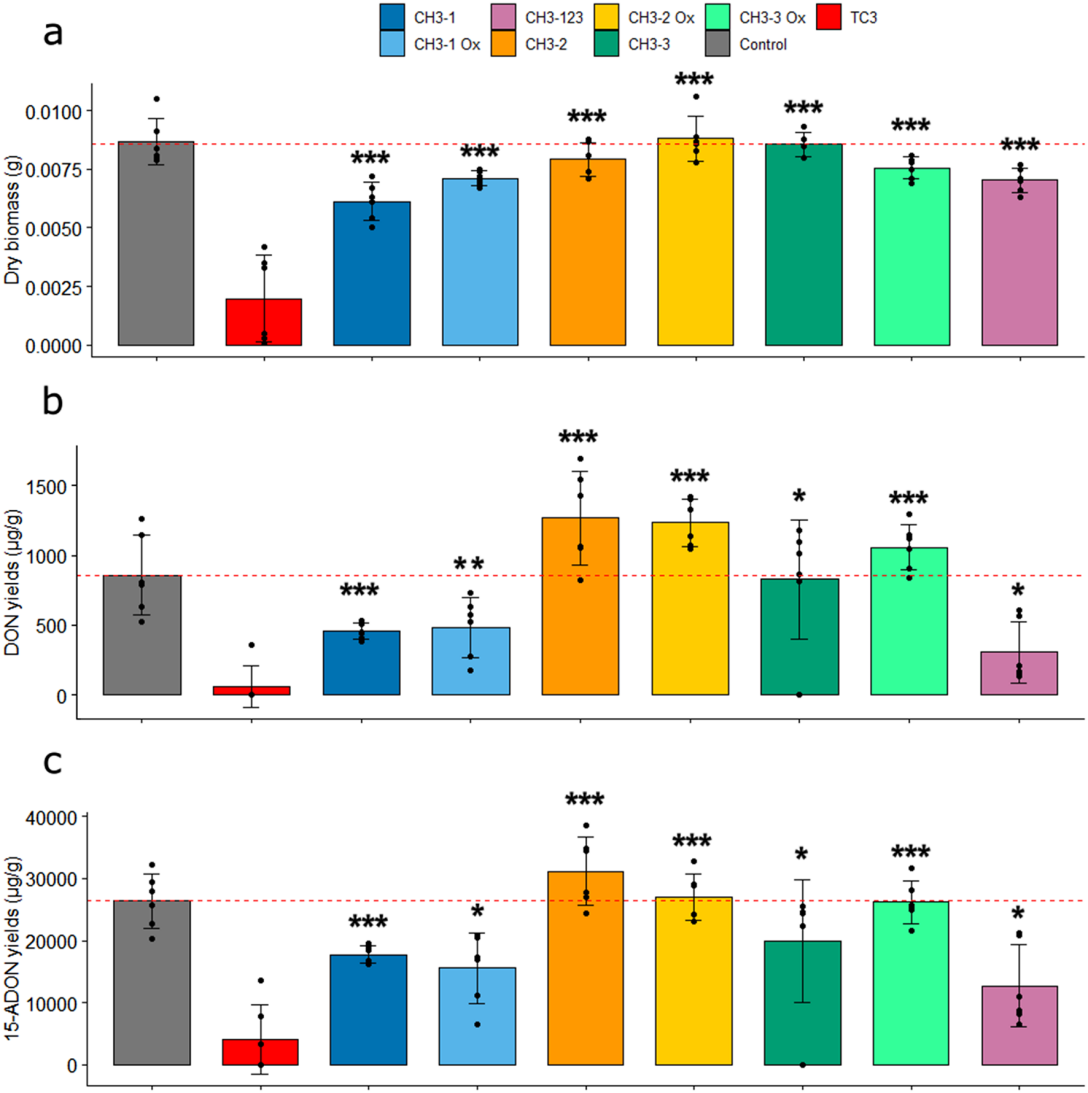
Antifungal and anti-mycotoxin activity of linear, methylated and oxidized TC3. Effect of reduced TC3, TC3-CH3-1, TC3-CH3-2, TC3-CH3-3, TC3-CH3-1 Ox, TC3-CH3-2 Ox, TC3-CH3-3 Ox and TC3-CH3-123 at 50 µM on the fungal biomass weight of *F. graminearum* (**a**) and on the production of DON (**b**) and 15-ADON (**c**) in 10-day-old broths. Significant differences are labelled (*p < 0.05, **p < 0.01 and ***p < 0.005).

All the methylated variants TC3-CH_3_-1, TC3-CH_3_-2, TC3-CH_3_-3 and the cyclized variants TC3-CH_3_-1Ox, TC3-CH_3_-2Ox, TC3-CH_3_-3Ox had significantly less effect on fungal mycelium growth when compared with TC3 (Fig. 4a). Mycotoxin production of *F. graminearum* was then measured after adding the different methylated-reduced and methylated-oxidized-cyclized variants at 50 µM. All variants had a significantly lower inhibitory effect on DON and 15-ADON production compared with TC3. However, the treatments with TC3-CH_3_-1 and TC3-CH_3_-1Ox led to a reduction in DON (Fig. 4b) and 15-ADON (Fig. 4c) levels compared to the control.

Thus, the production of 15-ADON was reduced by about 33% in presence of TC3-CH_3_-1 (*p* value = 0.002) and by about 42% in presence of TC3-CH_3_-1Ox (*p* value = 0.017). An additional peptide, TC3-CH_3_-123, with all Cys capped with CH_3_ groups was synthetized. *F. graminearum* exposed to TC3-CH_3_-123 produced significantly more DON and 15-ADON than TC3. The reduction in the ability to inhibit TCTB production was similar in all methylated-reduced and methylated-oxidized-cyclized variants, suggesting that Cys capping reduces the activity of TC3, regardless of oxidation and cyclization status. However, methyl caps on Cys9 (TC3-CH_3_-2Ox) and Cys11 (TC3-CH_3_-3Ox) had the most negative impact in the activity of TC3.

### The positively charged residues are essential for the antifungal and anti-mycotoxin activities of TC3

**Figure 5:**
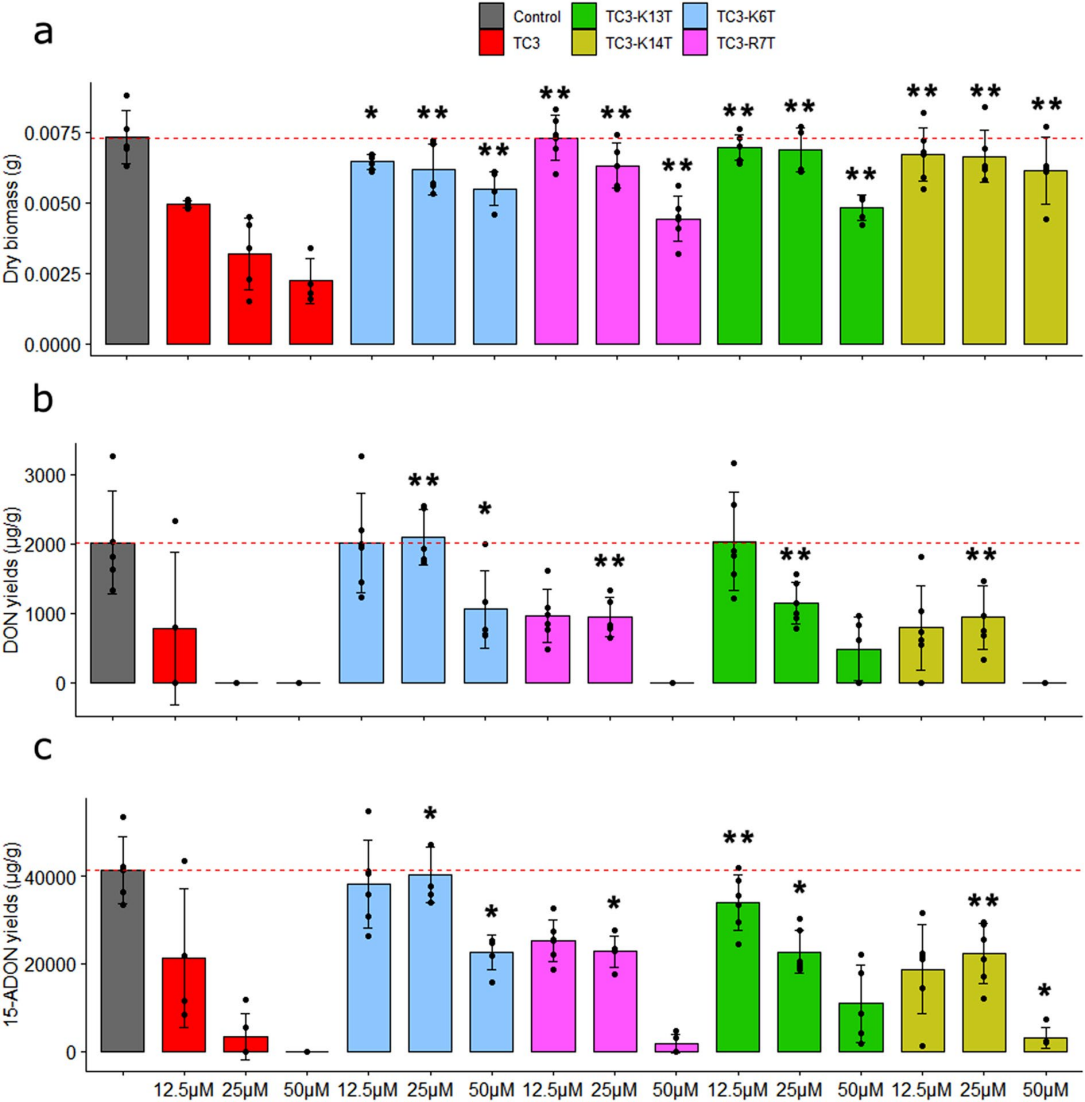
Role of positively charged amino acids on the activity of TC3. Effect of reduced TC3, TC3-K6T, TC3-R7T, TC3-K13T or TC3-K14T at 12.5, 25 and 50 µM on the fungal biomass weight of *F. graminearum* (**a**) and on the production of DON (**b**) and 15-ADON (**c**) in 10-day-old broths. Significant differences are labelled (*p < 0.05, **p < 0.01 and ***p < 0.005).

The role of cationic charges on the biological activity of TC3 was tested using TC3 variants in which positive residues Lys6 (TC3-K6T), Arg7 (TC3-R7T), Lys13 (TC3-K13T) and Lys14 (TC3-K14T) were individually substituted by the neutral amino acid Thr. Purity (*i.e*., ∼95%) and sequence of the TC3 variants were confirmed by HPLC and ESI–MS, respectively. The variants TC3-K6T, TC3-R7T and TC3-K13T had significant effect on fungal growth at 50 µM, when compared to the control (with respective p-values of 0.012, 0.004 and 0.012), while TC3-K14T had no effect at the tested concentrations. However, the antifungal activity of all variants was significantly reduced in comparison with that of native peptide TC3 (Fig. 5a).

Mycotoxin production by *F. graminearum* was measured in media supplemented with TC3-K6T, TC3-R7T, TC3-K13T and TC3-K14T at three different concentrations 12.5, 25 and 50 µM. All variants showed a lower DON (Fig. 5b) and 15-ADON (Fig. 5c) production inhibition activity compared with the native peptide TC3. Peptide TC3-K6T had the lowest inhibitory activity on TCTB production, suggesting that Lys6 plays important roles in the anti-mycotoxin activity of TC3.

## Discussion

The current study demonstrates that the γ-core motif TC3 reduces the growth of *F. graminearum* strain CBS 185.32 in a dose-dependent manner. In agreement with our findings, TC3 was previously reported to decrease germination in *F. graminearum* strain 8/1 with little or no effect on bacterial growth^9^. It is noteworthy that while DefMT3 has both antibacterial and antifungal effects^9^, TC3 has only antifungal effect suggesting that this γ-core is responsible for the antifungal activity of DefMT3. This also suggests that the structural bases of the antifungal and antibacterial activity of DefMT3 are independent. Previous studies showed that the γ-core of the plant defensin MtDef4 alone was capable of inhibiting *F. graminearum* growth, but not that of MsDef1^33^. Remarkably, 50 µM of TC3 reduced fungal growth 1.8 times and abrogated the production of mycotoxins DON and 15-ADON by *F. graminearum*. The moderate antifungal effect and high anti-mycotoxin effect suggest that the mechanism of inhibition of toxin biosynthesis is independent from the fungicide effect.

Arthropod defensins have six cysteine residues that form a conserved pattern of disulfide bridges in the combinations Cys1–Cys4, Cys2–Cys5 and Cys3–Cys6^34,35^. The formation of disulfide bridges by Cys cyclization confers stability to insect and chelicerate defensins^34,35^. Consequently, cyclization is expected to enhance the activity of defensins. For example, while both fully-oxidized and linear persulcatusin, a defensin from *Ixodes persulcatus* ticks, showed activity against *Staphylococcus aureus*, the activity of fully-oxidized peptides was significantly higher than that of linear peptides^36^. In addition, loss of a single disulphide bridge was enough to disrupt the antibacterial activity of Coprisin, a defensin of the beetle *Copris tripartitus*^34^. TC3 contains the last three Cys of DefMT3 (Cys4, Cys5 and Cys6-named in this study Cys1, Cys9 and Cys11 considering their position in TC3) and oxidation of TC3 forms three possible disulfide bridge combinations (i.e. Cys1–Cys9 or Cys1–Cys11 or Cys9-Cys11). None of these monomeric disulfide combinations was observed when oxidation was induced in the non-methylated TC3. Instead of oxidized monomers, a TC3 dimer (TC3–TC3) was detected by mass spectrometry suggesting that inter-molecular, and not intra-molecular, disulfide bridges prevail in spontaneous oxidative folding of TC3. The dimerization decreased the antifungal activity of TC3, contrasting with results obtained on plant defensin NaD1 where dimerization enhances its antifungal activity^37^. Antifungal activity assays showed that cyclic TC3 peptides have a severely reduced capacity to inhibit the fungal growth and the production of TCTB, compared with the linear TC3. This is in contrast with previous reports on human defensins in which cyclization enhanced the antimicrobial activity of γ-cores^38,39^.

Antifungal peptides were originally considered as lytic molecules acting primarily by permeabilizing the plasma membrane after interactions with cell wall components. Recently published data have indicated the preference of some defensins to interact with sphingolipids and phospholipids contained in lipid rafts, that house critical proteins for the regulation of membrane potential, intracellular pH, and nutrient transport^40,41^. Protein-lipid overlay assays showed that the plant defensin Psd2 targets the phospholipid POPC in the membrane of *Fusarium solani*^41^. In contrast to Psd2, our membrane modelling showed that TC3 is recruited by POPS, POPA and POPG, but not by POPC. Further studies using *F. graminearum* mutants lacking membrane phospholipids POPS, POPA, POPG or POPC could be used to test in vitro or in silico results. Besides, our results suggest that the four cationic acids contained in TC3 are partly responsible for its antifungal activity. Particularly, results show that Lys6 plays a key role in the anti-mycotoxin activity of TC3. The importance of Lys6 may be due to its positive charge combined with its position close to the Phe28 and Leu29 in the native protein DefMT3, which were predicted to interact with a lipid bilayer membrane in silico^10,42^. The role of cationic charges in the activity of antimicrobial peptides has been reported^43^. These cationic charges have been shown to be involved in the interaction of defensins with the negatively charged phospholipids of the fungal membrane leading to pore formation, dysregulation of ion homeostasis and cell death^44^.

Moreover, there are currently several evidences that antifungal peptides, including plant defensins, can reach intracellular targets and exert their activity by mechanisms in addition to permeabilization of the membrane^45,46^. In particular, these mechanisms involve the production of reactive oxygen species (ROS) in fungal cells and the inhibition of substrate acidification^47^, which are two environmental factors with a recognized modulating effect on the production of TCTB^48^. Defensins also interact with components of the tricarboxylic acid (TCA) cycle and GABA shunt^49^ that are tightly related with the mycotoxin biosynthesis pathway^50^. Despite being derived from a tick defensin, the mechanistic action of TC3 may be similar to that of insect antimicrobial peptides (AMP). For example, the AMP metchnikowin from *Drosophila melanogaster* has potent activity against F. graminearum^49^. Metchnikowin affects *F. graminearum* by inhibiting the activity of the fungal enzyme iron-sulfur subunit of succinate-coenzyme Q reductase, a key enzyme in the TCA cycle and the electron transport chain^49^. Alternatively, TC3 may specifically inhibit the activity of some enzymatic components of the TCTB biosynthetic pathway, in the same way that some phenolic compounds inhibit the activity of trichodiene synthase, the first and key enzyme of the cascade leading to the accumulation of TCTB^51^.

## Conclusion

TC3 is a potent antifungal peptide that inhibits germination^9^ and does not require Cys cyclization to inhibit the growth and TCTB production of *F. graminearum*. TC3 cyclization reduces the antifungal activity, especially when disulphide bridges are formed between Cys1-Cys11 or Cys1-Cys9. This suggests structural rigidity of the N-terminus having Cys1 reduced the activity of TC3. The cationic amino acids are partly responsible for the antifungal and anti-mycotoxin activities of TC3, notably Lys6. The results reported here advance our current understanding of the mechanism of action of tick molecules with antifungal and anti-mycotoxin activities and pave the way for developing novel and environment-friendly strategies for the control of *F. graminearum* using arthropod-derived peptides.

## Supplementary Information

Supplementary Figures.
