# Supplementary material for: Tick defensin γ-core reduces Fusarium graminearum growth and abrogates mycotoxins production with high efficiency
Source: Sci Rep. 2021 Apr 12;11:7962. doi: 10.1038/s41598-021-86904-w (PMC8042122; doi:10.1038/s41598-021-86904-w)
Supplement: Supplementary file 1 — Supplementary Figures. [file 41598_2021_86904_MOESM1_ESM.pdf]

Supporting Information

Tick defensin γ-core reduces Fusarium graminearum growth and abrogates mycotoxins production with high efficiency

Valentin Leannec-Rialland[1,†], Alejandro Cabezas-Cruz[2,†,*], Vessela Atanasova[3], Sylvain Chereau[3], Nadia Ponts[3], Miray Tonk[4,5,6], Andreas Vilcinskas[4,5,6,7], Nathalie Ferrer[3], James J Valdés[7-9] and Florence Richard-Forget[3,*]

[1]Université de Bordeaux, INRAE, Mycology and Food Safety (MycSA), F-33882 Villenave d'Ornon, France

[2]Anses, INRAE, Ecole Nationale Vétérinaire d'Alfort, UMR BIPAR, Laboratoire de Santé Animale, Maisons-Alfort, F-94700, France

[3]INRAE, Mycology and Food Safety (MycSA), F-33882 Villenave d'Ornon, France

[4]Institute for Insect Biotechnology, Justus Liebig University of Giessen, Heinrich-Buff-Ring 26-32, 35392 Giessen, Germany.

[5]LOEWE Centre for Translational Biodiversity Genomics (LOEWE-TBG), Senckenberganlage 25, 60325 Frankfurt, Germany.

[6]Fraunhofer Institute for Molecular Biology and Applied Ecology, Department of Bioresources, Ohlebergsweg 12, 35392 Giessen, Germany.

[7]Faculty of Science, University of South Bohemia, Branišovská 1160/31, 37005 České Budějovice, Czech Republic.

[8]Institute of Parasitology, Biology Centre, Czech Academy of Sciences, Branišovská 1160/31, 37005 České Budějovice, Czech Republic.

[9]Department of Virology, Veterinary Research Institute, Hudcova 70, 62100 Brno, Czech Republic.

†These authors contributed equally to this work

*Correspondance: Alejandro Cabezas-Cruz, PhD. Email: alejandro.cabezas@vet-alfort.fr. Florence Richard-Forget, PhD. Email: florence.forget@inrae.fr

Supplementary Figure S1. Experimental quantification of purity and determination of molecular weight for TC3 and TC3Ox using HPLC and ESI-MS, respectively.

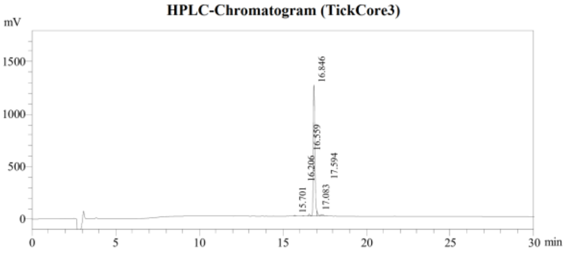

PeakTable

Peak	Ret. Time	Area	Height	Area %
1	15.701	35029	7964	0.394
2	16.206	9997	2738	0.112
3	16.559	107243	18503	1.206
4	16.846	8454978	1247647	95.082
5	17.083	280510	20828	3.155
6	17.594	4578	1532	0.051
Total		8892335	1299212	100.000

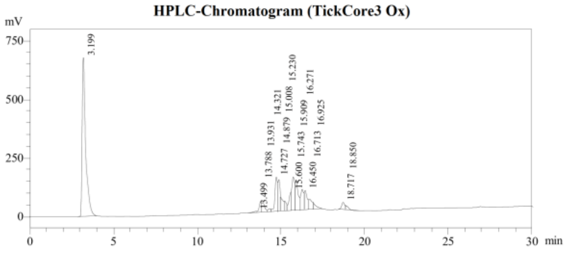

PeakTable

Peak	Ret. Time	Area	Height	Area %
1	3.199	9905530	676661	44.525
2	13.499	74933	4778	0.337
3	13.788	447158	42557	2.010
4	13.931	458022	40414	2.059
5	14.321	164691	12525	0.740
6	14.727	1554025	145507	6.985
7	14.879	1203875	134948	5.411
8	15.008	513869	56571	2.310
9	15.230	443616	40255	1.994
10	15.600	649509	77708	2.920
11	15.743	1724939	142109	7.754
12	15.909	1523004	125126	6.846
13	16.271	1060808	87085	4.768
14	16.450	992373	81448	4.461
15	16.713	651887	43308	2.930
16	16.925	322892	27976	1.451
17	18.717	355431	29789	1.598
18	18.850	200368	17092	0.901
Total		22246931	1785858	100.000

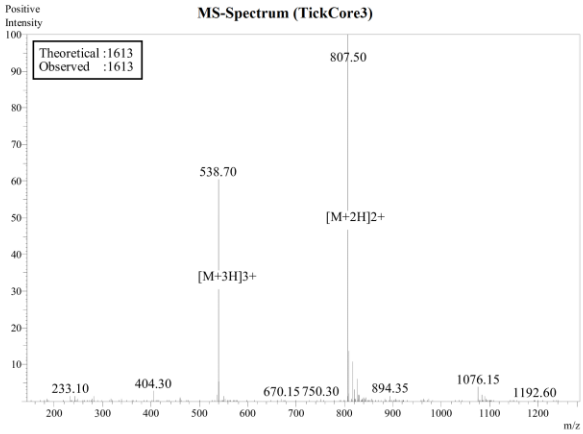

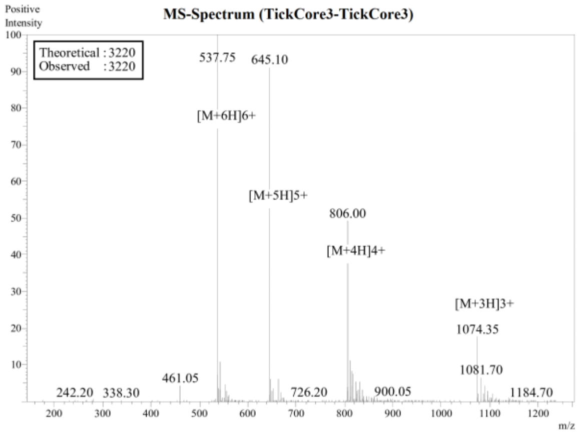

Supplementary Figure S2. TC3 interactions with POPS. The line graph is the distance measured (y-axis) between the α-carbon (αC) of TC3 peptide residues and the phosphorus (P) atom of POPS throughout the 1μs of MD (x-axis; in ns).

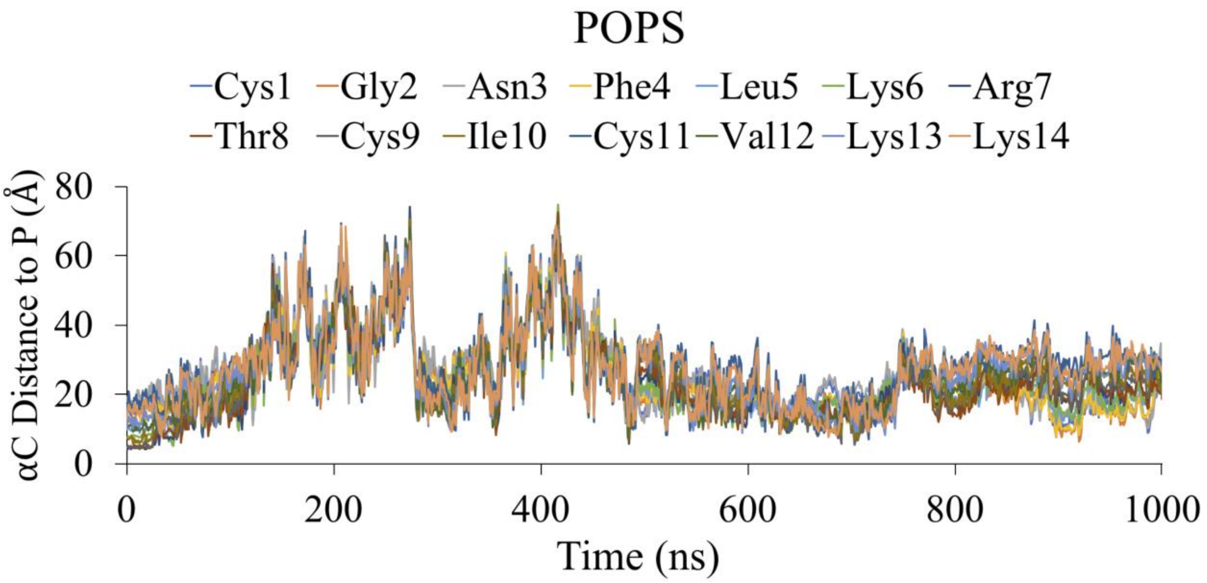

Supplementary Figure S3. TC3Ox interactions with POPA, POPG and POPS. The line graphs (A) are the distances measured (y-axis) between the α-carbon (αC) of TC3Ox residues and the phosphorus (P) atom of POPA, POPG and POPS for the 1μs of MD (x-axis; in ns). The secondary structure folds (legend; B) for each TC3OX peptide residue (y-axis) for the 1μs of MD (x-axis; in ns). The secondary structure graph was generated using the Timeline plug-in of the Visual Molecular Dynamics program (VMD)[49].

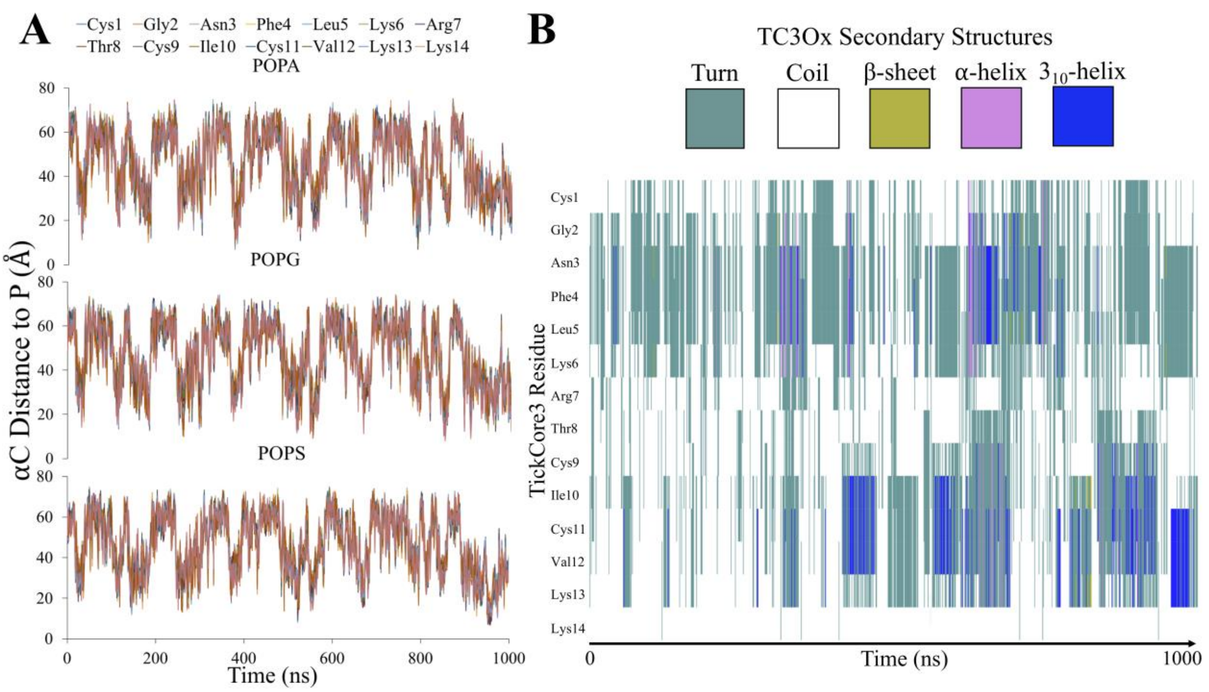

Supplementary Figure S4. TC3 secondary structure analysis. The graph depicts the secondary structure folds (legend) for each residue of the TC3 peptide (y-axis) throughout the 1μs of MD (x-axis; in ns). The graph was generated using the Timeline plug-in of the Visual Molecular Dynamics program (VMD)[49].

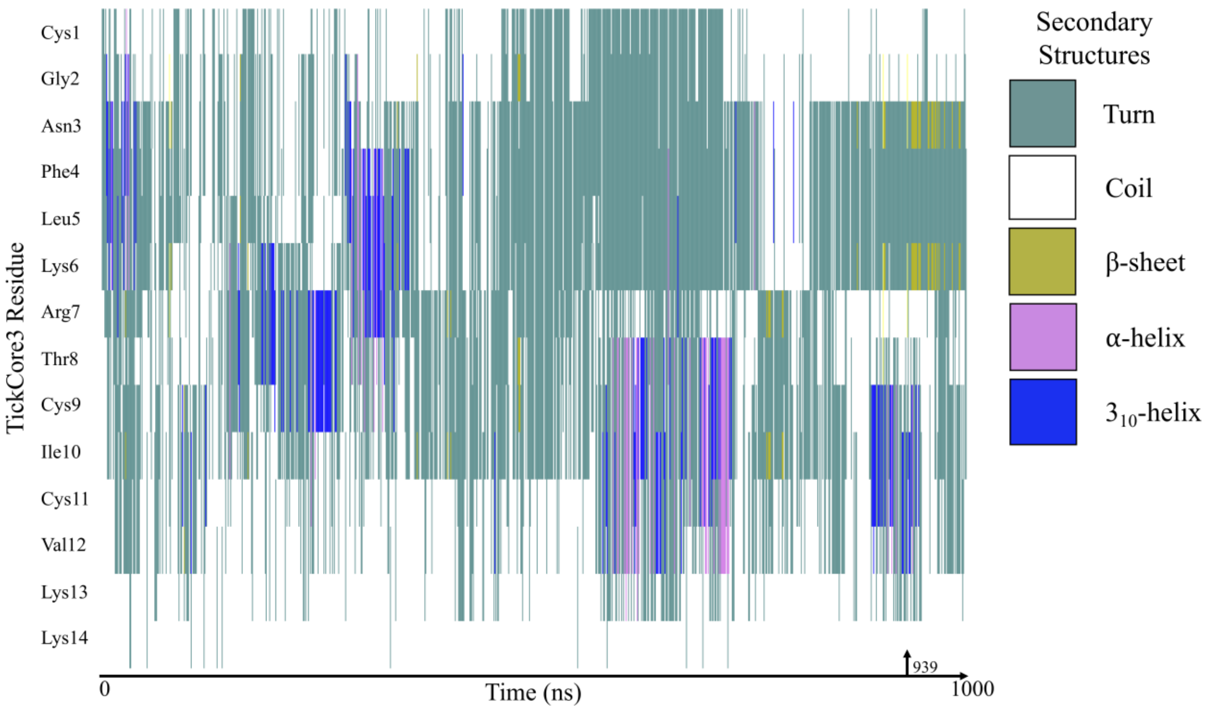

Supplementary Figure S5. Experimental quantification of purity and determination of molecular weight for TC3-CH3-1, TC3-CH3-2, TC3-CH3-3, TC3-CH3-1Ox, TC3-CH3-2Ox and TC3-CH3-3Ox using HPLC and ESI-MS, respectively.

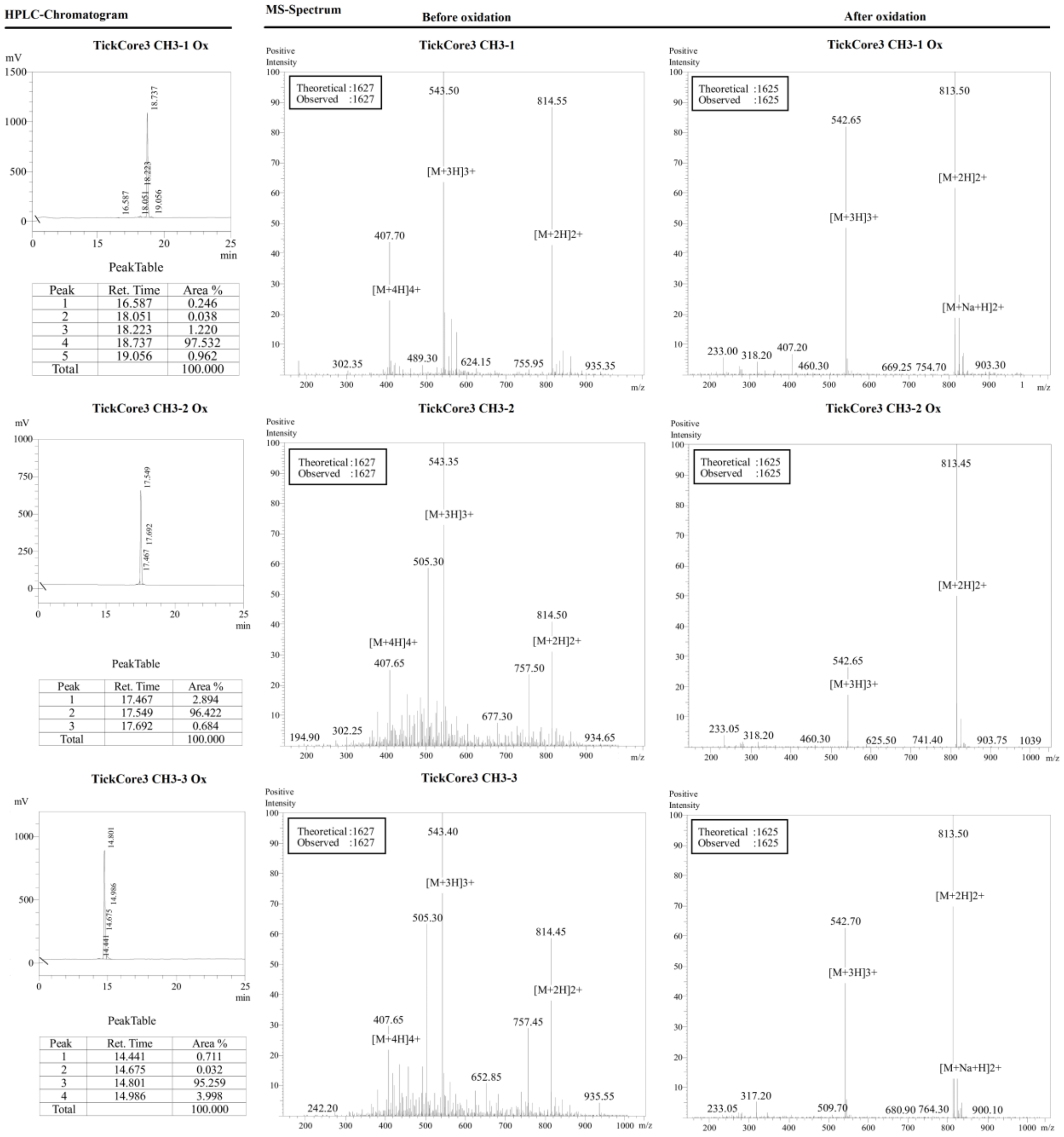

Peak	Ret. Time	Area %
1	16.587	0.246
2	18.051	0.038
3	18.223	1.220
4	18.737	97.532
5	19.056	0.962
Total		100.000

Peak	Ret. Time	Area %
1	17.467	2.894
2	17.549	96.422
3	17.692	0.684
Total		100.000

Peak	Ret. Time	Area %
1	14.441	0.711
2	14.675	0.032
3	14.801	95.259
4	14.986	3.998
Total		100.000
